# Biochemical Composition, Nutritional Profile, Bioactive Compound Content, Antioxidant Activity, and Technological Potential of Mycelium from *Pleurotus ostreatus* (Shimeji)

**DOI:** 10.3390/molecules31162851

**Published:** 2026-08-15

**Authors:** Ygor Velloso Tavares, Davi Vieira Teixeira da Silva, Luiz Torres Neto, Ana Júlia Bento do Amaral, Adaelson Firmino da Silva Junior, Vania Margaret Flosi Paschoalin, Rosane Marina Peralta, Alex Graça Contato, Carlos Adam Conte-Junior

**Affiliations:** 1 Analytical and Molecular Laboratorial Center (CLAn), Institute of Chemistry (IQ), Federal University of Rio de Janeiro (UFRJ), Cidade Universitária, Rio de Janeiro 21941-909, RJ, Brazil; vellosoygor@gmail.com (Y.V.T.); luiz-torres-neto@hotmail.com (L.T.N.); anajuliabentoamaral@gmail.com (A.J.B.d.A.); adaelsonf@gmail.com (A.F.d.S.J.); paschv@iq.ufrj.br (V.M.F.P.); conte@iq.ufrj.br (C.A.C.-J.); 2 Center for Food Analysis (NAL), Technological Development Support Laboratory (LADETEC), Federal University of Rio de Janeiro (UFRJ), Cidade Universitária, Rio de Janeiro 21941-598, RJ, Brazil; 3 Laboratory of Advanced Analysis in Biochemistry and Molecular Biology (LAABBM), Department of Biochemistry, Federal University of Rio de Janeiro (UFRJ), Cidade Universitária, Rio de Janeiro 21941-909, RJ, Brazil; davivieira@ufrj.br; 4 Graduate Program in Biochemistry (PPGBq), Institute of Chemistry (IQ), Federal University of Rio de Janeiro (UFRJ), Cidade Universitária, Rio de Janeiro 21941-909, RJ, Brazil; 5 Graduate Program in Food Science (PPGCAL), Institute of Chemistry (IQ), Federal University of Rio de Janeiro (UFRJ), Cidade Universitária, Rio de Janeiro 21941-909, RJ, Brazil; 6 Graduate Program in Chemistry (PGQu), Institute of Chemistry (IQ), Federal University of Rio de Janeiro (UFRJ), Cidade Universitária, Rio de Janeiro 21941-909, RJ, Brazil; 7 Department of Pharmaceutical Technology, Federal Fluminense University (UFF), Dr. Mario Vianna Street, 523, Niterói 24210-141, RJ, Brazil; 8 Department of Biochemistry, State University of Maringá, Maringá 87020-900, PR, Brazil; rmperalta@uem.br; 9 Department of Agricultural, Livestock and Environmental Biotechnology, Faculty of Agricultural and Veterinary Sciences (FCAV), Sao Paulo State University (UNESP), Jaboticabal 14884-900, SP, Brazil; 10 Graduate Program in Veterinary Hygiene (PPGHV), Faculty of Veterinary Medicine, Fluminense Federal University (UFF), Niteroi 24220-000, RJ, Brazil

**Keywords:** antioxidant properties, fungal biomass, phenolic compounds, texture profile analysis, edible mushroom, sustainable biotechnology

## Abstract

*Pleurotus ostreatus* (shimeji) is an edible mushroom widely recognized for its nutritional value and production of bioactive compounds. In addition to its fruiting body, mycelial biomass has emerged as a promising and sustainable alternative to produce bioactive compounds under controlled cultivation conditions. This study evaluated the nutritional composition, texture profile, biochemical composition, phenolic compounds, and antioxidant properties of the mycelium of *P. ostreatus*. Nutritional parameters were determined by FoodScan™ (NIR), while biochemical composition was assessed through spectrophotometric quantification of soluble proteins, free amino acids, reducing sugars, and total carbohydrates. Texture Profile Analysis (TPA) was performed to characterize the mechanical and technological properties of the mycelial biomass. Total phenolic compounds were quantified using the Folin–Ciocalteu method, and individual phenolics were identified by HPLC-DAD. Antioxidant activity was evaluated by ABTS, DPPH, TEAC, and hydroxyl radical scavenging assays. The mycelium exhibited high moisture content and relevant protein levels, as well as a rigid structural profile characterized by elevated hardness and gumminess. Biochemical characterization revealed considerable concentrations of soluble proteins and free amino acids, together with detectable levels of reducing sugars and total carbohydrates. The aqueous extract showed high total phenolic content and expressive antioxidant activity, with gallic acid identified as the predominant phenolic compound, followed by ferulic acid. The extracts also demonstrated efficient radical scavenging capacity in ABTS, DPPH, and hydroxyl radical assays. These findings highlight *P. ostreatus* mycelium as a sustainable source of bioactive compounds with potential applications as a functional ingredient in food, nutraceutical, and biotechnology sectors, reinforcing its role within circular bioeconomy strategies.

## 1. Introduction

The increasing consumer demand for natural, sustainable, and health-promoting products has stimulated the exploration of novel sources of bioactive compounds for applications in the food, nutraceutical, pharmaceutical, and biotechnology industries [1]. In this context, fungi have attracted scientific and industrial attention due to their remarkable metabolic diversity, nutritional richness, and ability to produce a wide range of biologically active molecules [2].

Edible fungi, commonly known as mushrooms, as well as their mycelial biomass, have emerged as promising sources of functional ingredients for foods and other value-added products [3]. In addition to their nutritional potential, these organisms contain bioactive compounds associated with antioxidant, anti-inflammatory, antimicrobial, immunomodulatory, and antitumor activities [4].

Edible mushrooms represent an important yet underexploited food source. Their cultivation is considered an efficient biotechnological strategy for converting lignocellulosic residues into protein-rich biomass, while simultaneously promoting the valorization of agro-industrial by-products and mitigating environmental impacts [5,6].

Among edible mushrooms, *Pleurotus ostreatus* (shimeji) stands out as one of the most widely cultivated and consumed species worldwide due to its high productivity, adaptability to different cultivation substrates, and low production cost [7]. This species is a rich source of proteins, essential amino acids, dietary fiber, β-glucans, vitamins, minerals, phenolic compounds, and several secondary metabolites associated with important biological activities [8].

**Figure 1:**
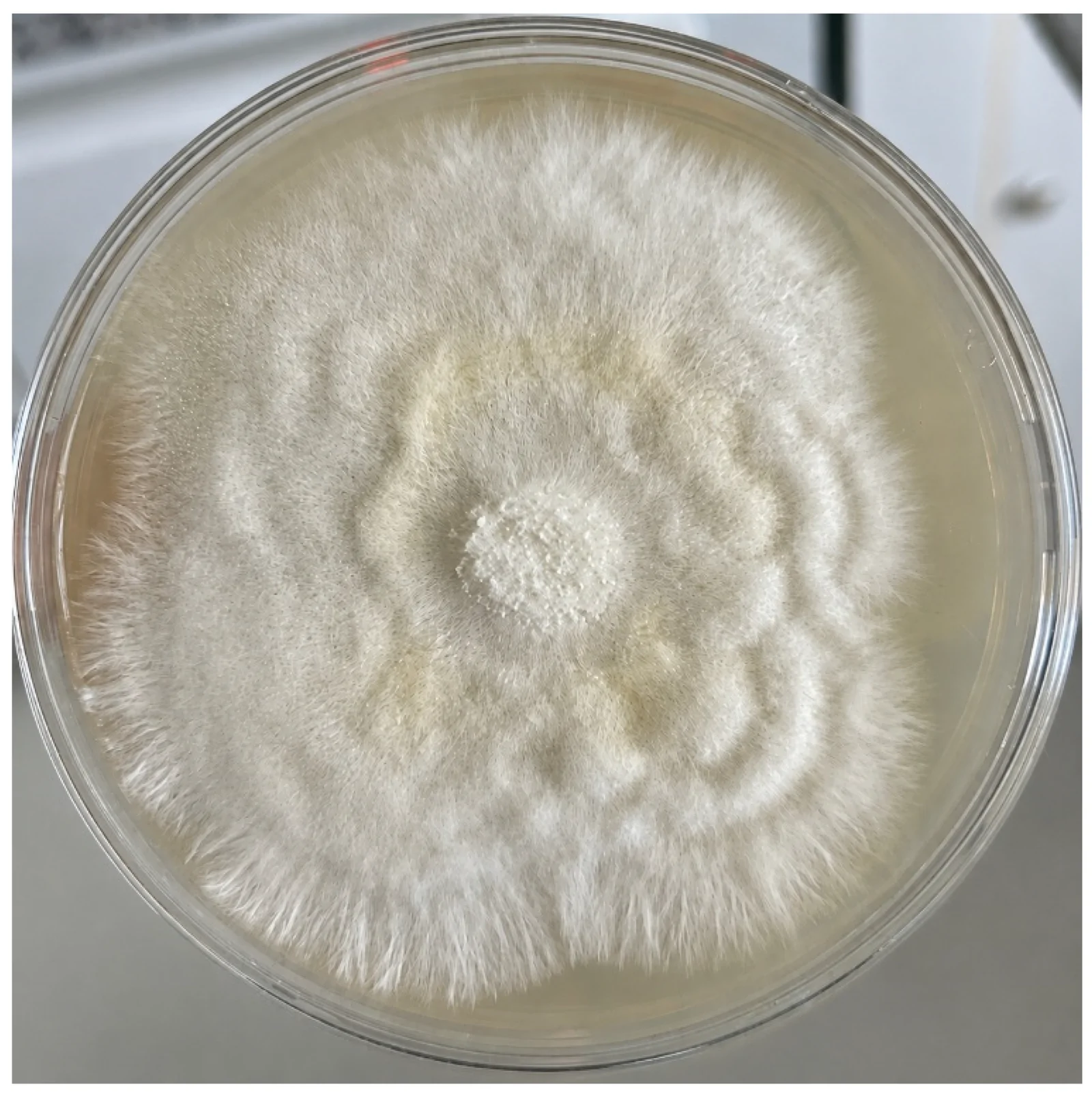
*Pleurotus ostreatus* mycelium cultivated on potato dextrose agar (PDA).

Interest in fungal mycelium, defined as the vegetative stage consisting of a network of hyphae responsible for nutrient absorption and metabolite production, has increased significantly over the past decades. Mycelium can also be cultivated under controlled conditions, allowing for greater standardization of growth parameters and chemical composition, while generally requiring shorter production cycles and being independent of fruiting body formation. These characteristics highlight mycelium as a promising and scalable raw material for food, nutraceutical, and biotechnological applications [9]. However, *P. ostreatus* mycelium (Figure 1) stands out for its nutritional and functional profile, containing dietary fiber, proteins, β-glucans, phenolic compounds, flavonoids, B-complex and C vitamins, and minerals, which are directly associated with antioxidant, immunomodulatory, hypocholesterolemic, and antimicrobial properties. Furthermore, this biomass exhibits a high metabolic capacity for the synthesis of extracellular enzymes, including laccases, peroxidases, cellulases, xylanases, and proteases, which are widely exploited in industrial processes such as fermentation [10].

The biochemical composition of mycelium is directly influenced by the fungal species, cultivation substrate, environmental conditions, and extraction methods, resulting in significant variations in the contents of proteins, free amino acids, polysaccharides, carbohydrates, and phenolic compounds [11]. Among these constituents, phenolic compounds, including phenolic acids and ergothioneine, stand out for their antioxidant capacity, acting as electron or hydrogen donors to neutralize reactive oxygen species and thereby reducing oxidative damage in biological systems [1]. This mechanism is particularly relevant in the context of diseases associated with chronic oxidative stress, such as cardiovascular disorders, type 2 diabetes, neurodegenerative diseases, and cancer [12]. In parallel, the filamentous structure of mycelium promotes the efficient colonization of lignocellulosic substrates and agro-industrial residues, enabling its application in bioremediation processes and the development of biomaterials and functional foods within the framework of a circular economy [13].

Despite the growing interest in fungal mycelium, important gaps remain in the characterization of *P. ostreatus* mycelium as a food and biotechnological raw material [13]. In particular, limited information is available regarding the integrated characterization of its nutritional composition, soluble protein and free amino acid contents, phenolic profile, and antioxidant potential in aqueous extracts, while most studies have focused primarily on the fruiting bodies [2]. Moreover, the relationship between the chemical and bioactive composition of *P. ostreatus* mycelium and its potential technological applications remains insufficiently explored.

Based on these considerations, we hypothesized that *P. ostreatus* mycelial biomass would exhibit a nutritionally relevant composition, including proteins, free amino acids, carbohydrates, and other bioactive constituents, together with measurable antioxidant potential. We further hypothesized that the aqueous extract would contain detectable phenolic compounds and that the complementary analytical approaches used in this study would allow the nutritional, biochemical, physicochemical, and functional characteristics of the mycelium to be comprehensively characterized.

Therefore, to test these hypotheses, the present study aimed to provide a comprehensive characterization of *P. ostreatus* mycelial biomass by evaluating its biochemical composition, soluble proteins, free amino acids, reducing sugars, total carbohydrates, and textural properties. Furthermore, the phenolic composition and antioxidant potential of an aqueous mycelial extract were investigated using antioxidant assays and HPLC-DAD analysis. By integrating these nutritional, biochemical, physicochemical, and antioxidant aspects, this study sought to clarify the potential of *P. ostreatus* mycelium as a sustainable source of functional ingredients and bioactive compounds for food and biotechnological applications.

## 2. Materials and Methods

### 2.1. Chemicals

Analytical standards, including gallic, caffeic, ferulic, p-coumaric, chlorogenic, and vanillic acids, as well as Folin–Ciocalteu reagent, Trolox (6-hydroxy-2,5,7,8-tetramethylchroman-2-carboxylic acid), DPPH (1,1-diphenyl-2-picrylhydrazyl), ABTS (2,2′-azinobis(3-ethylbenzothiazoline-6-sulfonic acid)), ninhydrin, Bradford reagent, and bovine serum albumin (BSA), were obtained from Sigma-Aldrich (St. Louis, MO, USA). HPLC-grade acetonitrile was purchased from Vetec (Duque de Caxias, RJ, Brazil), while ultrapure water was produced using a Milli-Q water purification system (Millipore, Bedford, MA, USA).

### 2.2. Sample Preparation

Fresh fruiting bodies of shimeji mushroom (*Pleurotus ostreatus*) were purchased from a local market in Rio de Janeiro, Brazil. The mycelial biomass used throughout this study was established from the internal tissue of the commercially obtained fruiting bodies. The mushrooms were aseptically cleaned, and small fragments of the internal tissue were excised under sterile conditions. One tissue fragment was transferred onto Petri dishes containing Potato Dextrose Agar (PDA) and incubated at 28 °C until a pure axenic culture was obtained. The resulting culture was subsequently used as the source of mycelial inoculum for biomass production.

Pure cultures of *P. ostreatus* grown on Potato Dextrose Agar (PDA) plates were used to inoculate Erlenmeyer flasks by transferring mycelial discs into a sterile liquid medium consisting of 20 g of wheat bran suspended in 100 mL of distilled water as described by Contato et al. [14] and supplemented with Vogel’s minimal medium at a 1:50 ratio [15]. Erlenmeyer flasks (250 mL) containing 50 mL of culture medium were incubated under constant agitation at 120 rpm and 28 °C for 5 days. After this period, the mycelial biomass was transferred to fresh culture medium of the same composition and incubated under the same conditions for an additional 7 days. Subsequently, the resulting mycelial biomass was recovered by filtration through Whatman no. 1 filter paper (Sigma-Aldrich, St. Louis, MO, USA), under aseptic conditions.

For aqueous extraction, approximately 1 g of mycelial biomass was suspended in 25 mL of sterile distilled water. The samples were maintained under agitation at 120 rpm and 28 °C for 1 h. Vacuum filtration was then performed using Whatman no. 1 filter paper, and the procedure was repeated three times to maximize the recovery of water-soluble compounds. The resulting filtrates were pooled, frozen, and lyophilized for 48 h.

The extraction yield was calculated after the lyophilization process based on the ratio between the mass of the lyophilized extract and the initial mass of the sample. Subsequently, the lyophilized extracts were stored at −20 °C until further analysis. Prior to biochemical characterization, phenolic compound determination, and antioxidant activity assays, the lyophilized extracts were reconstituted in deionized water at a concentration of 100 mg/mL. All subsequent analyses were performed using these reconstituted extracts. All procedures were performed using three independent biological replicates (*n* = 3).

### 2.3. Texture Profile Analysis (TPA)

Texture characterization of the samples was performed using the Texture Profile Analysis (TPA) method with a texture analyzer coupled to Exponent Connect 7.0 software and equipped with a cylindrical compression probe. TPA is a widely used instrumental technique that simulates the mechanical action of mastication through two successive compression cycles, allowing for the determination of several parameters related to the textural behavior of biological materials and food systems [16].

The samples consisted of *P. ostreatus* mycelial biomass cultivated on a solid culture medium in Petri dishes. For sample preparation, approximately 20 mL of culture medium was added to each Petri dish before inoculation, providing a standardized substrate thickness for mycelial development. After the cultivation period, the mycelial biomass remained naturally attached to the agar medium. The agar substrate was intentionally maintained throughout the analysis because its removal could damage the mycelial network and alter its native three-dimensional structure, consequently affecting its mechanical behavior. Therefore, texture measurements were performed on the intact mycelium–agar system, preserving the structural organization developed during cultivation. Each experimental treatment was evaluated in triplicate.

Texture measurements were performed directly on the mycelium-agar system. Prior to testing, the mycelium–agar matrix was carefully removed from the Petri dish and positioned at the center of the instrument platform to ensure proper alignment between the sample surface and the compression probe. The assay was conducted in double-compression mode, using a pre-test speed of 1.0 mm/s, test speed of 1.0 mm/s, and post-test speed of 1.0 mm/s. Samples were compressed to 40–50% of their original thickness, with a 5-s interval between compression cycles. The trigger force was set at 5 g to establish initial contact between the probe and the sample surface while minimizing pre-deformation effects [17].

The two compression cycles generated force–time and force–deformation curves that were automatically recorded by the Exponent Connect 7.0 software. From these curves, the main TPA parameters were determined, including hardness, springiness, cohesiveness, gumminess, chewiness, and resilience. Hardness was defined as the maximum force reached during the first compression cycle. Springiness, cohesiveness, and resilience were calculated from the distances and areas under the force curves generated during the test. Gumminess was calculated as the product of hardness and cohesiveness, whereas chewiness was determined as the product of gumminess and springiness [16,18].

All analyses were performed under controlled experimental conditions, including prior equipment calibration and standardized sample preparation procedures, to ensure the accuracy, reliability, and reproducibility of the obtained data [18].

### 2.4. Nutritional Profile

The nutritional composition of the samples was determined by Near-Infrared Spectroscopy (NIR) using a FoodScan™ analyzer (Foss Analytics, Hillerød, Denmark). The evaluated parameters included moisture, crude protein, fat, ash, and salt contents. This analytical approach is based on calibration models previously developed from conventional reference methods, enabling the rapid and reliable simultaneous quantification of multiple constituents [19].

Near-infrared spectroscopy (NIR) has been widely employed in food characterization due to its non-destructive nature, minimal sample preparation requirements, and high analytical speed. The technique is based on the interaction of radiation in the near-infrared region (780–2500 nm) with the sample matrix, where absorption occurs primarily through overtone and combination vibrations of molecular bonds such as O–H, C–H, and N–H. The spectral information obtained is subsequently processed using chemometric calibration models, which correlate spectral responses with compositional data generated by standardized analytical reference methods [19].

The use of the FoodScan™ system enabled the rapid determination of the nutritional composition of the samples, providing accurate and reliable results while significantly reducing the time required for analysis compared with conventional laboratory methods.

### 2.5. Biochemical Characterization

#### 2.5.1. Total Protein

Protein content was determined using the Bradford method [20] with Coomassie Brilliant Blue G-250 dye (Sigma-Aldrich, St. Louis, MO, USA). The extracts were mixed with the Bradford reagent and incubated for 5 min at room temperature in the dark. The interaction between the reagent and proteins resulted in a spectrophotometric change measured at 595 nm. Quantification was performed using a bovine serum albumin (BSA) standard curve, and the results were expressed as mg/mL of bovine serum albumin (BSA) equivalents.

#### 2.5.2. Free Amino Acids

Free amino acids were quantified using the ninhydrin method, which reacts with free amino groups to form colored compounds. The extracts were mixed with ninhydrin solution and heated under controlled conditions. After cooling, absorbance was measured at 570 nm. The concentration was determined using an alanine standard curve, and the results were expressed as μg/mL of alanine equivalents [21].

#### 2.5.3. Reducing Sugars

Reducing sugar content was determined using a colorimetric method based on the reaction with 3,5-dinitrosalicylic acid (DNS). Aliquots of the extracts (500 μL) were mixed with the DNS reagent and heated in a water bath for a predetermined period, promoting reagent reduction and the formation of a colored complex. After cooling, absorbance was measured at 540 nm. Quantification was performed using a glucose standard curve, and the results were expressed as μg/mL of glucose equivalents [22].

#### 2.5.4. Total Carbohydrates

Total carbohydrate content was estimated using the anthrone method. The extracts were treated with anthrone reagent, prepared by adding 5 mL of absolute ethanol to a 100 mL flask, dissolving 200 mg of anthrone, and bringing the volume to 100 mL with 75% (*v*/*v*) sulfuric acid. This procedure promotes the hydrolysis and dehydration of sugars, resulting in the formation of colored compounds. After the addition of the anthrone reagent, the samples were heated in a water bath for 10 min and subsequently cooled in an ice bath. Following incubation, absorbance was measured at 625 nm. Quantification was performed using a glucose standard curve, and the results were expressed as μg/mL of glucose equivalents [23].

### 2.6. Antioxidant Activity

#### 2.6.1. Abts and Teac-Abts

The antioxidant capacity of the extracts was evaluated using the ABTS•^+^ radical assay, according to a methodology adapted from the literature. The radical was previously generated by the reaction between an ABTS solution (2,2′-azinobis(3-ethylbenzothiazoline-6-sulfonic acid), 7 mM) and potassium persulfate (2.45 mM) and allowed to stand for 12–16 h in the dark at room temperature. Prior to analysis, the solution was diluted with methanol (59 mL) until an absorbance of approximately 1.1 ± 0.02 at 734 nm was achieved. Subsequently, an aliquot of the extract (7.5 μL) was added to the radical solution (142.5 μL) and incubated for 2 h under controlled conditions. Absorbance was monitored spectrophotometrically, and antioxidant activity was expressed as EC_50_, defined as the concentration required to neutralize 50% of the radical [24]. Total antioxidant capacity was also expressed as Trolox Equivalent Antioxidant Capacity (TEAC). The values obtained were interpolated using a Trolox standard curve and expressed as μmol of Trolox equivalents per gram of sample (μmol TE/g) [25].

#### 2.6.2. DPPH and TEAC-DPPH

The free radical scavenging activity was determined based on the ability of the extracts to reduce the stable radical 2,2-diphenyl-1-picrylhydrazyl (DPPH•) [26]. For this purpose, a DPPH solution (0.1 mM in methanol) was prepared and mixed with the extract. After incubation in the dark for 1 h, the decrease in absorbance was measured at 517 nm. The results were expressed as EC_50_ values. In addition, Trolox Equivalent Antioxidant Capacity (TEAC-DPPH) was determined, and the results were expressed as μmol of Trolox equivalents per gram of sample (μmol TE/g).

#### 2.6.3. Hydroxyl Radical Scavenging Capacity

Hydroxyl radical scavenging activity was evaluated through radical generation based on the Fenton reaction, which is widely applied in oxidative processes. The reaction mixture contained ferric ions, hydrogen peroxide, and salicylic acid, which acts as a chromogenic agent sensitive to oxidation [27].

The presence of antioxidant compounds in the extracts promoted the inhibition of oxidative product formation, and absorbance was measured at a wavelength of 510 nm [28]. The results were expressed as mM of ascorbic acid equivalents per mg of extract.

### 2.7. Bioactive Compound Content

#### 2.7.1. Total Phenolic Compounds

The total phenolic content was determined using the Folin–Ciocalteu reagent. Aliquots of the extracts (1 mL) were mixed with Folin–Ciocalteu reagent (1 N), previously diluted in distilled water at 1:1 (*v*/*v*), followed by the addition of sodium carbonate solution [29]. After incubation for 1 h at room temperature in the dark, absorbance was measured at 725 nm. The results were calculated using a calibration curve constructed with gallic acid and expressed as micrograms of gallic acid equivalents per gram of sample (µg GAE/g).

#### 2.7.2. HPLC-DAD Analysis of Polyphenols

The identification and quantification of individual phenolic compounds were performed by high-performance liquid chromatography (HPLC) using an LC-20-A Prominence system (Shimadzu^®^, Kyoto, Japan) equipped with a diode array detector (DAD). Separation was performed according to Ulusu et al. [30] using a Nucleosil C18 column (5 µm, 150 × 4.6 mm) with the column temperature maintained at 25 °C under gradient elution using different mobile phases—(A) 2% acetic acid (*v*/*v*); (B) acetonitrile with acetic acid (99.5:0.5, *v*/*v*); and (C) pure acetonitrile—at a flow rate of 1.0 mL min^−1^.

The elution program was set as follows: 90% A and 10% B from 0 to 30 min; 80% A and 20% B from 30 to 60 min; 55% A and 45% B from 60 to 62 min; and 40% B and 60% C from 62 to 77 min. At the end of the chromatographic run, the initial conditions (90% A and 10% B) were restored from 77 to 80 min to allow for column re-equilibration [31]. These conditions were maintained for an additional 5 min to ensure system stabilization before initiating the subsequent analysis. The injection volume was 10 µL.

Detection of the compounds was performed at 280 and 320 nm. The diode array detector enabled simultaneous monitoring of analytes across different spectral regions, increasing analytical selectivity and aiding in the identification of phenolic compounds present in the samples. Quantification was performed by external calibration using analytical standards prepared in methanol at a concentration of 100 µg mL^−1^.

The preparation of the extracts was performed with adaptations of the methodology described by Hu et al. [31]. Approximately 100 mg of sample was extracted with methanol at a 1:35 (*m*/*v*) ratio, corresponding to 3.5 mL of solvent. Extraction was performed in an ultrasonic bath at 40 °C for 40 min. Subsequently, the samples were centrifuged, and the resulting supernatants were collected and directly used for chromatographic analyses. The choice of methanol as the extraction solvent was based on previously reported results by Ulusu et al. [30], which demonstrated higher efficiency in recovering phenolic compounds compared to aqueous extraction systems.

### 2.8. Statistical Analysis

All experiments were performed using three independent biological replicates (*n* = 3). Each biological replicate consisted of an independently cultivated *P. ostreatus* mycelium grown in a separate Petri dish containing the same culture medium, identical supplementation, and the same initial biomass under identical incubation conditions. Each biological replicate was processed independently throughout all experimental procedures, from biomass collection to biochemical analyses.

Results are expressed as mean ± standard deviation (SD). Statistical analyses and graphical representations were performed using GraphPad Prism^®^ version 9.0 (GraphPad Software, San Diego, CA, USA). EC_50_ values were determined by linear interpolation using the software. Scatter plots were generated using the same software.

## 3. Results and Discussions

### 3.1. Texture Profile Analysis (TPA)

Shimeji (*P. ostreatus*) mycelium consists of a three-dimensional (3D) network of interconnected hyphae, primarily composed of structural polysaccharides such as chitin and β-glucans, as well as proteins and other fungal cell wall components [3]. This composition directly influences its physical and mechanical properties, making mycelial biomass an interesting material with technological potential for applications in food systems, biotechnology, and the development of sustainable biomaterials [32]. Texture characterization is essential to understand its structural behavior and suitability for different applications [33].

In this context, Texture Profile Analysis (TPA) was employed to determine parameters such as hardness, springiness, cohesiveness, gumminess, chewiness, and resilience, which provide information on the material’s response to mechanical deformation and its ability to maintain structural integrity after the application of external forces [33]. The results obtained for *P. ostreatus* mycelium are presented in Table 1.

Based on the results presented in Table 1, *P. ostreatus* mycelium exhibited high values of hardness (3948.56 ± 681.40) and gumminess (81.44 ± 22.95) when compared to the other parameters, namely springiness (0.03 ± 0.01), cohesiveness (0.02 ± 0.01), chewiness (1.77 ± 0.29), and resilience (0.02 ± 0.01). Overall, these results indicate a material with high initial resistance to compression, but low structural recovery capacity after deformation and reduced internal integrity among the mycelial hyphae [34].

The high hardness observed (3948.56 ± 681.40 g) suggests that the mycelial matrix formed by *P. ostreatus* presents a compact structure resistant to compression. This behavior can be attributed to the presence of a 3D hyphal network enriched in chitin and β-glucans, structural components known to confer mechanical rigidity to mycelium [33]. Recent studies report that increased hyphal network density significantly enhances the mechanical strength of mycelium-based materials, particularly in systems developed from *P. ostreatus* [34,35].

The springiness value was extremely low (0.03 ± 0.01), indicating that after compression the sample recovered only a small fraction of its original height. This result indicates predominantly inelastic behavior, characteristic of fibrous and highly structured materials. The low springiness may be related to hyphal organization and the limited mobility of the polymeric network formed by chitin and glucans [36]. Recent studies on *P. ostreatus* mycelial materials highlight that springiness strongly depends on hyphal network architecture and the water content within the matrix [33,37].

Cohesiveness also showed a low value (0.02 ± 0.01), indicating that the internal structure of the material undergoes relatively easy rupture after the first compression cycle [37]. Thus, although the material is rigid, it has a limited ability to withstand repeated deformations without structural loss. Low cohesiveness values are commonly observed in biological systems composed of highly porous fibrous networks, in which deformation leads to the disruption of interactions between fibers or hyphae [38,39].

The gumminess value of 81.44 ± 22.95 results from the product of hardness and cohesiveness. Despite the high hardness, the moderate gumminess value was limited by the low cohesiveness observed. This suggests that the mycelium requires considerable force to be initially deformed but exhibits reduced structural resistance after the onset of rupture [33]. This behavior is consistent with mycelial materials in which mechanical resistance is concentrated in a compacted surface network, while the internal structure exhibits higher porosity.

Chewiness showed a value of 1.77 ± 0.29, which is relatively low when compared to the material’s hardness. Since this parameter depends on both gumminess and springiness, the low springiness values directly contributed to the reduced energy required to continue deformation after the initial rupture [40]. In food systems, low chewiness values are typically associated with materials that fragment rapidly during mastication, without exhibiting significant elastic behavior.

Finally, resilience (0.02 ± 0.01) was significantly low, indicating an inability of the material to recover the energy applied during the first compression cycle. Studies on biomaterials produced from *P. ostreatus* show that resilience tends to decrease as hyphal network densification and structural rigidity increase [38,40,41].

Overall, the obtained textural profile characterizes *P. ostreatus* mycelium as a rigid, low-springiness, low-cohesiveness structure with limited recovery after deformation, a behavior consistent with the formation of a three-dimensional network rich in chitin and β-glucans [41]. These characteristics suggest that *P. ostreatus* mycelium may represent a promising source of biomolecules for future investigations into food-related and biomaterial applications. However, studies evaluating its technological performance and functional properties in specific application systems are required to substantiate these potential uses [40].

### 3.2. Nutritional Profile

**Figure 2:**
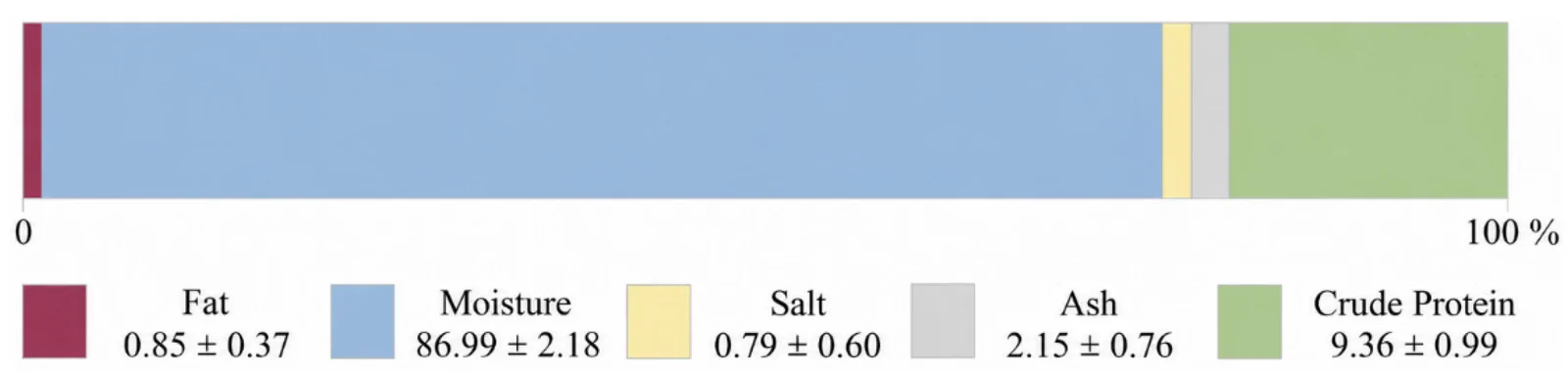
Nutritional composition of the mycelium of shimeji (*P. ostreatus*), expressed as percentage (%).

The nutritional composition of *P. ostreatus* mycelium showed high moisture content compared to crude protein, lipids, ash, and salt content (0.79 ± 0.60%), as presented in Figure 2. These results evidence a nutritional profile consistent with that described for mycelial biomass of edible fungi, characterized by high water content, low lipid concentration, and the presence of nutritionally relevant compounds [42].

The high moisture content observed is a common characteristic of fresh fungi and mycelial biomass, directly influencing technological aspects such as microbiological stability, preservation, and processing. Similar values have been reported for *P. ostreatus*, with moisture content close to 91% in fresh samples, indicating that the biomass produced in this study exhibits behavior comparable to that described in the literature [43]. Ahmed et al. [44] reported that the nutritional composition of *P. ostreatus* is strongly influenced by the cultivation substrate, although high water retention remains a consistent characteristic of the species. This high moisture content is associated with the fungal cell wall structure, rich in β-glucans, chitin, and other hydrophilic polysaccharides, which promote water retention and contribute to the technological properties of the biomass.

The crude protein content (9.36 ± 0.99%) confirms the nutritional potential of mycelium as an alternative protein source. Although this value is lower than those reported for dry biomass of *P. ostreatus*, which frequently presents concentrations above 17% and may exceed 20% depending on cultivation conditions and the substrate used, this difference is expected due to the high moisture content of the analyzed sample [43]. Recent studies highlight that the protein composition of mycelium is strongly influenced by nitrogen source availability and the fermentation conditions employed during cultivation [45,46].

The low lipid content (0.85 ± 0.37%) is consistent with the nutritional profile generally attributed to fungi of the genus *Pleurotus*, which are considered low-fat foods [46]. Values close to 1.21% have been reported for *P. ostreatus* fruiting bodies, while studies specifically addressing mycelium also describe concentrations below 1%, reinforcing the low lipid density characteristic of fungal biomass. This condition is often associated with the potential use of these organisms in food formulations aimed at low-fat dietary applications [46].

The ash value of 2.15 ± 0.76% indicates the presence of minerals in the biomass, although at a lower concentration than that reported in some studies on fruiting bodies and dehydrated mycelium [47,48]. Recent studies report values ranging from 3% to 8% for *P. ostreatus*, a variation mainly attributed to differences in cultivation substrate, nutrient medium formulation, and analytical methods employed [44]. Ash content represents an important indicator of the mineral fraction of the food, including elements such as potassium, magnesium, calcium, and iron, which are commonly found in edible mushrooms [49].

The salt content was 0.79 ± 0.60%, which is relatively low and a desirable characteristic for food applications. Although this parameter is less frequently reported in the literature for mycelial biomass, its value suggests a low natural sodium concentration in the material, favoring its use as an ingredient in food formulations aimed at reduced sodium content [50].

The nutritional composition observed in *P. ostreatus* mycelium is closely associated with its active metabolic state during vegetative growth. Unlike fruiting bodies, the mycelium continuously synthesizes structural polysaccharides, proteins, and intracellular metabolites required for hyphal extension and substrate colonization. Consequently, differences in protein, carbohydrate, and lipid contents between mycelium and basidioma are expected and may also reflect cultivation conditions, substrate composition, growth stage, and nutrient availability. These physiological factors strongly influence carbon partitioning and nitrogen assimilation, ultimately affecting the nutritional profile of the fungal biomass [49,50].

Recent investigations have shown that differences in biomass composition are not solely determined by fungal species but are also markedly influenced by cultivation practices. Variations in substrate formulation, environmental conditions, and genetic background have been associated with significant changes in protein content, polysaccharide accumulation, antioxidant capacity, and mineral composition of edible mushrooms. In *Auricularia cornea*, Khan et al. demonstrated considerable variability among strains regarding biological efficiency, polysaccharide concentration, antioxidant activity, and mineral profile, emphasizing that cultivation parameters play a decisive role in defining the nutritional and functional value of mushroom-derived products. Therefore, comparisons among studies should consider species-specific characteristics and differences in production systems and cultivation protocols [51].

Overall, the obtained nutritional composition indicates that *P. ostreatus* mycelium exhibits characteristics consistent with those described for edible fungi of biotechnological interest, standing out for its high moisture content, low lipid content, relevant protein levels, and mineral presence. In submerged cultivation, Hamza et al. [52] observed high simultaneous production of biomass and exopolysaccharides, while other authors have reported that mycelium exhibits significant antioxidant activity and immunomodulatory potential. These attributes reinforce the potential of mycelial biomass as a sustainable ingredient for food, nutraceutical applications, and the development of novel mycoprotein-based foods [42,53]. In addition, recent reviews highlight that fungal mycelium offers relevant environmental advantages, including lower land and water requirements compared to conventional animal protein production systems [52].

### 3.3. Extraction Yield of Mushroom Extracts

The efficiency of extracting bioactive compounds from fungal mycelium is an important parameter for evaluating the recovery of target metabolites and understanding the chemical composition of the obtained extracts. As observed for fruiting bodies, extraction yield from mycelium is influenced by factors such as fungal species, cultivation conditions, solvent used, temperature, and extraction time [54]. However, in mycelial biomass, yields obtained through aqueous extraction tend to be lower due to the high proportion of insoluble structural components, such as chitin and β-glucans associated with the cell wall, which exhibit low water solubility [27,54].

**Figure 3:**
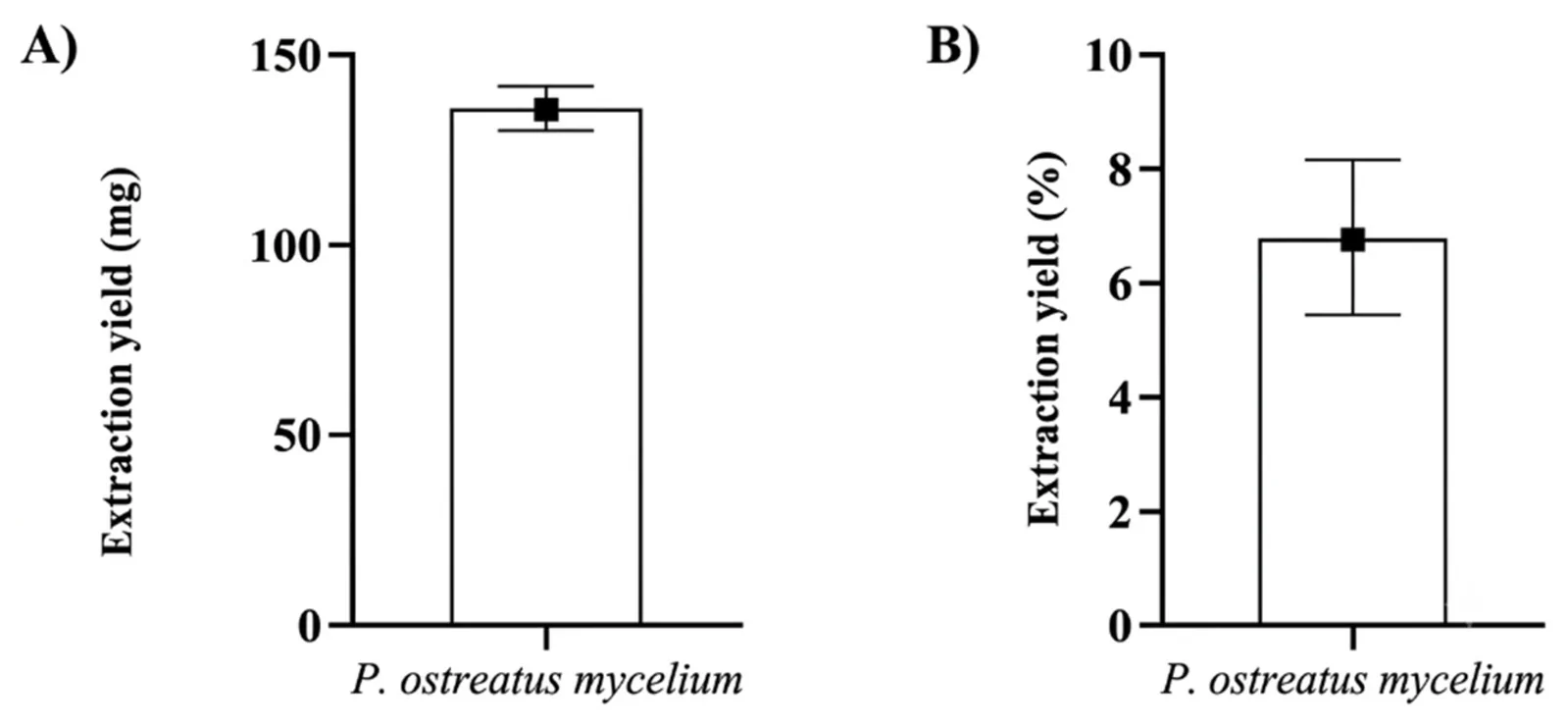
Yield of aqueous extracts of shimeji (*P. ostreatus*) mycelium after lyophilization. (**A**) Dry extract recovered from 2.0 g of fresh mycelial biomass (136.0 mg), corresponding to an extraction yield of 6.8%; (**B**) extraction yield expressed as a percentage based on the fresh weight of the mycelial biomass.

In the present study, 2 g of *P. ostreatus* mycelium was initially used to obtain the aqueous extract, yielding 136.0 mg of dry extract, corresponding to an extraction yield of 6.8% (Figure 3). It is important to note that the yield calculation was based on the fresh weight of the mycelial biomass. Thus, differences observed when compared to values reported in the literature may be related not only to the extraction conditions employed but also to the basis used for yield calculation, as many studies express their results relative to dry sample weight. Furthermore, the literature shows that yields obtained from mycelium are generally lower than those observed for fruiting bodies, due to structural and compositional differences between these two forms of fungal biomass. Mycelium consists of a network of hyphae rich in structural polymers, which remain largely associated with the cell wall matrix during conventional aqueous extraction, reducing the amount of solubilized and recovered material [27,55].

Although the obtained yield was relatively low, this result does not necessarily indicate a lower concentration of bioactive compounds. Aqueous extraction is known for its high selectivity toward polar metabolites, including soluble polysaccharides, proteins, peptides, and phenolic compounds, while a large fraction of insoluble constituents remain in the residual biomass. Recent studies have shown that more intensive methods, such as ultrasound-assisted extraction, microwave-assisted extraction, or specific chemical treatments, can significantly increase extraction yields from mycelium. Thus, the 6.8% yield observed in this study reflects both the selective nature of aqueous extraction and the characteristic structural composition of *P. ostreatus* mycelium [56,57].

### 3.4. Total Proteins, Free Amino Acids, Reducing Sugars, and Total Carbohydrates

The biochemical composition of aqueous extracts from *P. ostreatus* mycelium was evaluated by determining total proteins, free amino acids, reducing sugars, and total carbohydrates. Quantification of these constituents enabled the characterization of the mycelial biomass and provided support for the interpretation of results related to antioxidant activity and the functional potential of the species. The results obtained are presented in Table 2.

The results show that aqueous extracts from *P. ostreatus* mycelium contain significant amounts of total proteins, free amino acids, reducing sugars, and total carbohydrates, highlighting the chemical complexity of the produced mycelial biomass. Total protein content determined by the Bradford method [20] was 1099.27 ± 128.12, while free amino acid concentration reached 804.77 ± 138.36. These results indicate that the mycelium presents a composition rich in nitrogen-containing compounds, supporting the nutritional potential commonly attributed to species of the genus *Pleurotus* [58]. Recent studies have demonstrated that the protein composition of *P. ostreatus* can vary significantly depending on the substrate and cultivation conditions, reinforcing the influence of production factors on the nutritional quality of the biomass [57,58,59].

In addition to the protein fraction, reducing sugars (37.05 ± 7.139 µg glucose equivalents/mg extract) and total carbohydrates (26.85 ± 0.57 µg glucose equivalents/mg extract) were detected in the aqueous extract of *P. ostreatus* mycelium. These compounds are consistent with the typical chemical composition of fungal biomass, in which structural polysaccharides such as β-glucans and chitin, as well as other soluble carbohydrates, play important structural and functional roles. Although polysaccharides were not individually quantified in the present study, their potential contribution is relevant when interpreting the carbohydrate fraction and the antioxidant properties of the mycelial extract. In a comparative study of *Auricularia cornea* strains, Khan et al. [60] used FTIR spectroscopy to characterize functional groups associated with the molecular structure of the mushroom and identified signals attributed to β-glucans and polysaccharides, including bands around 1375 and 1402 cm^−1^ in the wild domesticated strain, as well as a band around 1025 cm^−1^ associated with C–O bonds in β-(1→3)-glucan and cell-wall polysaccharides in another commercial strain. These findings reinforce the importance of fungal polysaccharides as structural components of mushroom biomass and support the interpretation of the carbohydrate fraction observed in *P. ostreatus* mycelium. Moreover, polysaccharides and β-glucans have been widely recognized as bioactive fungal constituents that may contribute to antioxidant and other functional properties, although their specific contribution cannot be isolated from the present experimental design. Therefore, the carbohydrate content observed in *P. ostreatus* mycelium, together with its phenolic compounds and nitrogen-containing metabolites, may contribute to the multifunctional bioactive profile of the aqueous extract [46,59,60].

The combination of proteins, free amino acids, and carbohydrates observed in this study suggests that *P. ostreatus* mycelium represents a biomass of high biotechnological interest. Compared to the fruiting body, traditionally consumed as food, mycelium offers the advantage of being produced under controlled systems, allowing for greater standardization of chemical composition and a reduced time required for biomass production [46]. Thus, the results obtained reinforce the potential of mycelium as a raw material for the development of functional ingredients and value-added products intended for the food and nutraceutical sectors [61].

### 3.5. Antioxidant Properties and Total Phenolics

The antioxidant activity of aqueous extracts obtained from *P. ostreatus* mycelium was evaluated using different methods, including ABTS•^+^, DPPH• assays, Trolox equivalent antioxidant capacity (TEAC), and hydroxyl radical scavenging activity. In addition, total phenolic content was determined, as these metabolites are frequently associated with the antioxidant potential of edible fungi [54]. The use of different antioxidant methods is necessary because each assay is based on distinct mechanisms of action and exhibits variable sensitivity to the bioactive compounds present in the sample, enabling a more comprehensive and reliable assessment of the antioxidant capacity of the analyzed material [62]. The results obtained are presented in Table 3, allowing for the correlation between phenolic content and the observed antioxidant activity of the produced mycelium.

Aqueous extracts from *P. ostreatus* mycelium exhibited significant antioxidant activity across the different evaluated systems, demonstrating the ability to act through multiple mechanisms of reactive species neutralization. In the ABTS assay, an EC_50_ value of 77.29 ± 3.43 mg/mL and a Trolox equivalent antioxidant capacity (TEAC) of 4.49 ± 0.34 µmol TE/mg were observed, indicating efficiency in electron transfer for stabilization of the ABTS•^+^ radical. These results suggest that the bioactive compounds present in the extract can act in both hydrophilic systems and more complex environments [25].

In the DPPH assay, the extract showed an EC_50_ of 101.02 ± 1.39 mg/mL and a Trolox equivalent antioxidant capacity (TEAC) of 4.52 ± 0.11 µmol TE/mg. Although DPPH is considered a more selective and less reactive radical than ABTS, the results demonstrate that the mycelium contains compounds capable of donating hydrogen atoms and interrupting chain reactions associated with oxidation [54]. The differences observed between the ABTS and DPPH assays are expected due to the chemical specificities of each radical and the distinct mechanisms involved in antioxidant interactions [63].

In addition, the extract exhibited high hydroxyl radical scavenging capacity (71.21 ± 3.40 mM ascorbic acid equivalents per mg of extract), a particularly relevant result given the high reactivity of this oxidizing species. The hydroxyl radical is considered one of the most aggressive reactive oxygen species in biological systems, capable of causing damage to proteins, lipids, and nucleic acids. Thus, the high activity observed suggests the potential application of the mycelium as a functional ingredient with protective properties against oxidative processes [64]. The combination of high levels of phenolic compounds, especially gallic acid, and the presence of other bioactive metabolites such as proteins, amino acids, and polysaccharides, likely contributes to the overall antioxidant effect observed [52,54].

The antioxidant performance observed in *P. ostreatus* mycelium can be further contextualized by comparison with other edible mushrooms. Khan et al. [60], in a comparative evaluation of wild, domesticated, and commercially cultivated *A. cornea* strains, reported substantial differences in antioxidant activity among strains. The wild domesticated strain Ac24 exhibited the strongest DPPH radical-scavenging activity, with an EC_50_ of 0.23 mg/mL, whereas the commercial strains Ac3, Ac15, and Ac1 showed EC_50_ values of 0.8133, 1.0867, and 1.3733 mg/mL, respectively. In the FRAP assay, antioxidant capacity ranged from 266 to 591 mg Trolox equivalents/g, with Ac15 and Ac24 showing the highest values. Interestingly, Ac24 also showed 85% inhibition of AAPH-induced erythrocyte hemolysis, indicating that the antioxidant potential of *A. cornea* extended beyond chemical radical-scavenging assays. These findings are consistent with the antioxidant activity observed in the present *P. ostreatus* mycelial extract, although direct numerical comparisons should be interpreted cautiously because the studies used different fungal species, biological stages, extraction procedures, and antioxidant assays. Nevertheless, the variation among *A. cornea* strains demonstrates that antioxidant activity in mushrooms is strongly dependent on fungal genotype and production conditions, supporting the importance of considering cultivation conditions when interpreting the bioactive potential of *P. ostreatus* mycelium [60].

These results confirm that *P. ostreatus* mycelium represents a promising source of natural antioxidant compounds, with potential applications in the development of functional foods, nutraceutical ingredients, and formulations aimed at health promotion [65].

### 3.6. HPLC-DAD Analysis of Polyphenols

The phenolic composition of the *P. ostreatus* mycelial extract was investigated by high-performance liquid chromatography (HPLC) to identify the main compounds potentially associated with the observed antioxidant activity. Identification was based on retention times and characteristic wavelengths of analytical standards. The detected compounds and their respective concentrations are presented in Table 4.

Chromatographic analysis revealed that gallic acid was the main phenolic compound identified in the *P. ostreatus* mycelial extract, presenting a concentration approximately 12× higher than that of ferulic acid. The predominance of this compound suggests that it may significantly contribute to the antioxidant potential observed in the mycelium, since gallic acid is widely recognized for its high ability to donate electrons and hydrogen atoms [54]. The presence of gallic acid in *P. ostreatus* has been reported in several studies involving both fruiting bodies and cultivated mycelium and is frequently associated with high free radical scavenging capacity and protection against oxidative damage [66]. Structurally, this compound contains three hydroxyl groups attached to the aromatic ring, a feature that enhances its strong reducing activity and efficiency as a natural antioxidant. Thus, the concentration detected in this study may have contributed to the significant antioxidant activity values obtained in the ABTS and DPPH assays [67].

The high total phenolic content observed in the present study is also comparable, in terms of the overall occurrence of phenolic constituents in edible mushrooms, with the values reported for *A. cornea* by Khan et al. [60]. In their study, the total phenolic content ranged from 8.76 to 20.10 mg GAE/g of fruiting body, with the wild domesticated strain Ac24 exhibiting the highest value (20.10 mg GAE/g). The authors also observed substantial differences among strains, with commercial Ac3 and Ac15 presenting 17.23 and 12.53 mg GAE/g, respectively. This variability reinforces that phenolic accumulation in mushrooms is influenced by biological and production-related factors, including strain characteristics and cultivation conditions. In the present study, the predominance of gallic acid (0.51 ± 0.20 mg/g) over ferulic acid (0.04 ± 0.02 mg/g) suggests a specific phenolic profile associated with the mycelial growth conditions employed. Therefore, differences between *P. ostreatus* mycelium and *A. cornea* fruiting bodies should not be interpreted solely as species-specific effects, since the biological stage, cultivation substrate, extraction solvent, and analytical procedure may substantially influence phenolic recovery and composition.

On the other hand, ferulic acid was detected at a lower concentration. Despite its low level, its presence is relevant due to its well-documented antioxidant, anti-inflammatory, and antimicrobial properties in the literature [55,68]. Ferulic acid acts mainly through the stabilization of free radicals via electron resonance enabled by its methoxy and hydroxyl groups conjugated to the unsaturated side chain. In addition, this compound may act synergistically with other phenolics present in the extract, enhancing the overall antioxidant activity even when found at low concentrations [69]. The other phenolic acids were not found in the samples.

The results indicate that the antioxidant activity of the mycelium cannot be attributed exclusively to the identified phenolic compounds. Considering the high antioxidant capacity values observed in the chemical assays, it is likely that other bioactive metabolites present in the extract also contributed to the observed effect [69]. Species of the genus *Pleurotus* are known to produce a wide range of antioxidant compounds, including flavonoids, phenolic acids not identified by this method, ergothioneine, polysaccharides, β-glucans, bioactive peptides, and secondary metabolites, which may act individually or synergistically [67].

Another relevant aspect is that the phenolic profile of mycelium may differ substantially from that found in fruiting bodies. Factors such as culture medium composition, carbon and nitrogen sources, temperature, pH, incubation time, and physiological growth stage directly influence the biosynthesis of phenolic metabolites. Thus, the concentrations observed in this study reflect specific cultivation conditions and demonstrate that *P. ostreatus* mycelium constitutes a viable source of bioactive phenolic compounds, even in the absence of fruiting body formation [42,70].

The chromatographic data corroborate the results of the antioxidant assays and demonstrate that *P. ostreatus* mycelium exhibits a phenolic profile characterized by the predominance of gallic acid and the presence of ferulic acid in lower amounts. These compounds likely contribute to the observed antioxidant activity; however, the magnitude of the effect suggests the additional involvement of other bioactive metabolites produced by the fungus. These findings reinforce the biotechnological potential of mycelium as a sustainable source of natural antioxidants for applications in functional foods, nutraceuticals, and pharmaceutical products [45].

## 4. Conclusions

The present study demonstrated that *P. ostreatus* mycelium exhibits nutritional, biochemical, and functional characteristics that support its potential as a sustainable source of bioactive compounds. The mycelial biomass displayed a textural profile characterized by high hardness and gumminess, indicating a rigid structure with potential relevance for applications in the food and biotechnology industries. In addition, nutritional analysis revealed relevant protein content, low lipid levels, and the presence of minerals, while biochemical characterization showed considerable concentrations of soluble proteins, free amino acids, reducing sugars, and total carbohydrates, confirming the nutritional and functional value of this biomass.

The aqueous extract exhibited high total phenolic content and remarkable antioxidant activity, as demonstrated by the ABTS, DPPH, TEAC, and hydroxyl radical scavenging assays. HPLC-DAD analysis identified gallic acid as the predominant phenolic compound, followed by ferulic acid, indicating that these compounds, together with other bioactive metabolites present in the mycelium, may contribute to the antioxidant capacity observed.

Overall, the results demonstrate that *P. ostreatus* mycelium represents a promising biomass for further investigation as a source of functional ingredients, bioactive compounds, and value-added raw materials for potential applications in the food, nutraceutical, and biotechnology sectors. However, the potential for specific functional food, nutraceutical, or pharmaceutical applications requires further validation through biological, bioavailability, safety, and application-based studies. Beyond its nutritional and antioxidant properties, submerged cultivation of mycelial biomass offers advantages such as greater process standardization, shorter production cycles, and independence from fruiting body formation, making it a sustainable production strategy with potential alignment with the principles of the circular bioeconomy. Therefore, the findings of this study expand the current knowledge of the biotechnological potential of *P. ostreatus* mycelium and provide a scientific basis for future investigations aimed at optimizing cultivation and extraction processes, as well as identifying additional bioactive compounds and evaluating their bioavailability and biological effects.

## Figures and Tables

**Table 1:** Texture parameters of *Pleurotus ostreatus* mycelium.

Mycelium	Hardness	Springiness	Cohesiveness	Gumminess	Chewiness	Resilience
*P. ostreatus*	3948.56 ± 681.40	0.03 ± 0.01	0.02 ± 0.01	81.44 ± 22.95	1.77 ± 0.29	0.02 ± 0.01

Texture profile analysis (TPA) parameters of *P. ostreatus* mycelium. Values are expressed as mean ± standard deviation (SD) obtained from three independent biological replicates (*n* = 3). Hardness, gumminess, and chewiness are expressed in gram-force (g), whereas springiness, cohesiveness, and resilience are dimensionless parameters.

**Table 2:** Biochemical composition of aqueous extracts from the basidiome of shimeji (*P. ostreatus*).

Mycelium	Total Proteins —Bradford ^a^	Free Amino Acids ^b^	Reducing Sugar —DNS ^c^	Total Carbohydrates —Anthrone ^d^
*P. ostreatus*	1099.27 ± 128.12	804.77 ± 138.36	37.05 ± 7.14	26.85 ± 0.57

^a^ Results expressed in µg of BSA/mg of extract. ^b^ Results expressed in µg of alanine equivalents/mg of extract. ^c^ Results expressed in µg of glucose equivalents/mg of extract. ^d^ Results expressed in µg of glucose equivalents/mg of extract.

**Table 3:** Total phenolic content and antioxidant activities of aqueous extracts from *Pleurotus ostreatus* mycelium.

Mycelium	Phenolic Compounds ^a^	ABTS ^b^	TEAC ABTS ^c^	DPPH ^b^	TEAC DPPH ^c^	Hydroxyl Radical Scavenging ^d^
*P. ostreatus*	12,199.46 ± 1950.13	77.29 ± 3.43	4.69 ± 0.34	101.02 ± 1.39	4.52 ± 0.11	71.21 ± 3.40

^a^ Results expressed in µg of gallic acid equivalents/mg of extract. ^b^ Result expressed in EC_50_ (effective concentration) (mg/mL); ^c^ result expressed in µM of Trolox equivalent/mg of extract; ^d^ result expressed in mM of ascorbic acid equivalent/mg of extract.

**Table 4:** Phenolic compounds identified by HPLC-DAD in the *Pleurotus ostreatus* mycelium.

Phenolic Compound	Retention Time (min) ^a^	*P. ostreatus* Mycelium (mg/g) ^b^
Gallic acid (280 nm)	2.5	0.51 ± 0.2
Ferulic acid (320 nm)	35.0	0.04 ± 0.02

^a^ Retention time; ^b^ mg/g of extract.

## Data Availability

The original contributions presented in this study are included in the article. Further inquiries can be directed to the corresponding author.
